# Investigating Possible Dipole‐Bound States of Cyanopolyynes: the Case for the C_5_N^−^ Anion Detected in Interstellar Space

**DOI:** 10.1002/cphc.202300248

**Published:** 2023-10-24

**Authors:** Stanka Jerosimić, Milan Milovanović, Marko Mitić, Roland Wester, Francesco A. Gianturco

**Affiliations:** ^1^ University of Belgrade - Faculty of Physical Chemistry Stu-dentski trg 12–16 11158 Belgrade Serbia; ^2^ Institut für Ionenphysik und Angewandte Physik Universitaet Innsbruck Technikerstr. 25 A-6020 Innsbruck Austria

**Keywords:** multireference ab initio methods, ISM: molecular anions, dipole bound excited states, ISM: astrochemistry

## Abstract

We present results of quantum structure calculations aimed at demonstrating the possible existence of dipole‐bound states (DBS) for the anion $C_{5}N^{-}$ , a species already detected in the Interstellar medium (ISM). The positive demonstration of DBS existence using *ab initio* studies is an important step toward elucidating possible pathways for the formation of the more tightly bound valence bound states (VBS) in environments where free electrons from starlight ionization processes are known to be available to interact with the radical partner of the title molecule. Our current calculations show that such excited DBS states can exist in $C_{5}N^{-}$ , in agreement with what we had previously found for the smallercyanopolyyne in the series: the $C_{3}N^{-}$ anion. This system has a very weakly bound anion with binding energies of about 3 and 9 cm^−1^ for the 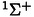 and 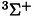 DBS, respectively.

## Graphical abstract

**Can the radical** C_5_N bind a free electron to form a dipole‐bound state? The answer is sought using multi‐reference *ab initio* quantum chemical methods.

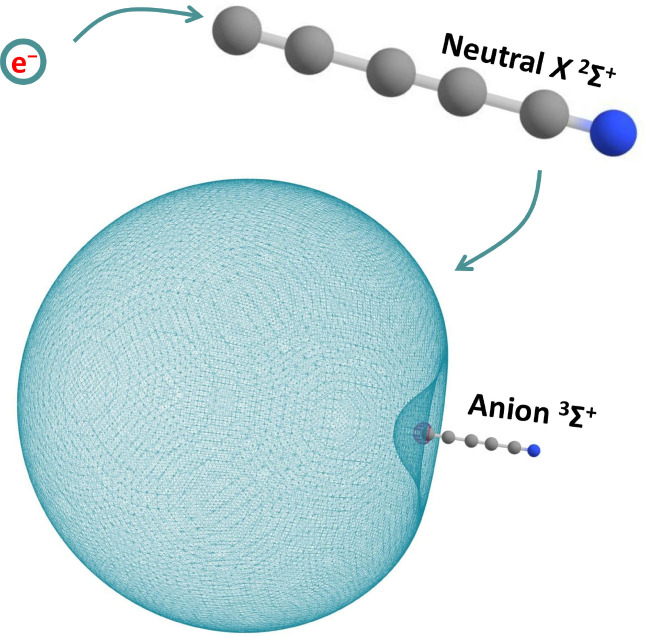

## Introduction

In recent years, several linear C‐bearing and (C,N)‐bearing chains of molecular anions have been detected at various sites in the interstellar medium (ISM) and circumstellar envelopes (CSE). Specifically: CN^−^,^[1]^ C_3_N^−^,^[2]^ C_5_N^−^,[^3^, ^4^] C_4_H^−^,[^5^, ^6^] C_6_H^−^,^[7]^ and C_8_H^−^.[^8^, ^9^] Those reported so far constitute different terms of the general linear chains associated with the polyynes and cyanopolyynes species. Very recently two additional (C,N)‐bearing chains have been detected that included the two largest chains of those series which have been observed so far: C_10_H^−[10]^ and C_7_N^−^.^[11]^

Astrophysical observation of molecular anions relies heavily on the spectroscopic investigations of their properties in the laboratory and on matching sighted lines with those observed in the earthly experiments. Additionally, to carry out astrophysical modeling of molecular population evolution from their distributions over a variety of internal states, important indicators are provided by the rate coefficients for the probability of rotational state‐changes in molecular species induced by their interaction with He and H_2_, both partners being present in substantial amounts in the ISM. The collision‐induced occurrence of non‐LTE (Local Thermal Equilibrium) population distribution for rotational states in molecular partners could, in fact, be a significant path to further radiative emission from different excited spectral lines in the observational microwave regions. Measurement of these rate coefficients in the laboratory is still challenging, while available quantum methods can be used to model such processes and then enter their results within networks of the kinetics of the underlying chemistry. For example, Botschwina and Oswald^[12]^ had computationally determined the structural characteristics of some of these species to help observational sighting in the ISM or in the laboratory.

The chemistry of formation of such cyanopolyyne chains has also been the object of several studies and speculations (e. g. see: Ref. [4, 13]).The possible formation of anions from the neutral radical via a Radiative Electron Attachment (REA) process has been considered in some detail (as quoted in: [14]) while a more direct chemical route by reaction of the HC_5_N with H^−^ has also been put forward by our group.^[15]^ Another possible mechanism of formation could be the reaction between N atom and C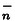 .^[4]^ For the present title molecule our calculated rates were found to be large enough to be relevant within the chemical evolution producing these anions. And as stated by Cernicharo et al.,^[4]^ the much larger rate constant of REA to C_5_N compared to C_3_N is the main reason why C_5_N^−^ is calculated to be much more abundant with respect to the neutral than C_3_N^−^ (the abundance ratios of the C_*n*_N /C_*n*_N^−^ are ~140 and 2.3 for n=3 and n=5 , respectively, in Taurus molecular cloud 1 (TMC‐1)). In the final analysis, however, the various options indicated by the current literature have not yet coalesced into a unique proof of the chemical formation of the title anion.

From the structural viewpoint, therefore, it is still of interest to be able to establish as reliably as possible whether or not the present cyanopolyyne chain can support a special class of anionic bound states which would then provide dynamical gateways to the stabilization of the more conventional, more strongly bound valence anions of the same molecular species. More specifically, and similarly to what has been found to occur for the smaller term in the same series, i. e. the C_3_N^−^ anion, and reported there from our previous studies,[^16^, ^17^, ^18^] we aim in the present study at establishing the existence of one or more Dipole‐Bound States (DBSs) for the larger term in the cyanopolyyne series, C_5_N^−^.

Previous studies on the possible dipole‐bound states of this anion came to different conclusions. Fortenberry,^[19]^ using the equation‐of‐motion coupled‐cluster (EOM‐CCSD) method, found that both C_3_N^−^ and C_5_N^−^ support a single dipole‐bound state. Later, by using the equation‐of‐motion coupled‐cluster method augmented with complex absorbing potential to capture metastable states,^[20]^ Fortenberry's earlier results were questioned as they found a very small dipole moment for the ground state of the neutral C_5_N. Although a series of low‐lying valence excited states with singlet and triplet spin symmetry were calculated, only dipole‐stabilized resonances were found in C_5_N^−^, but no dipole‐supported bound states. Structural studies on the anions of the HCN molecule were discussed in the earlier work from Simons’ group.^[21]^

Previous experimental studies were useful for this work, in particular the reported spectra of the C_5_N^−^ anion with time‐of‐flight photoelectron (TOF‐PE) spectroscopy, which provided an accurate electron detachment energy,^[22]^ then the first laboratory detection of the anion by microwave spectroscopy by Kasai et al.,^[23]^ and the recent detection of this neutral/anion pair in TMC‐1 mentioned above.^[4]^ As far as we are aware, there is no experimental information on the presence of any DBS in this anion. As already mentioned earlier, our experimental group in Innsbruck has recently published direct experimental evidence of the presence of DBS anions in the shorter C_3_N^−^ term of the series.^[16]^

This paper is organized as follows: the following Section, named The computational tools, outlines the *ab initio* methods used in our calculations for the title system. The next Section, named Results and Discussion, reports in detail the present results and discusses the implications of our computational findings for the possible formation of the bound anions in the title molecule. Finally, the conclusions are given in the last Section, named Present conclusions.

## The computational tools

The electronic structure calculations reported here were performed using the MOLPRO[^24^, ^25^, ^26^] and ORCA^[27]^ quantum chemistry program packages. Multi‐reference post‐Hartree‐Fock methods were used to calculate excited electronic states. Due to the existence of excited valence states of the anion^[20]^ with the same symmetry as the possible dipole bound states, calculations were performed for specific excited electronic configurations, using basis sets with very diffuse functions for the calculations of possible excited dipole bound states (DBXS).

To accurately describe the neutral C_5_N, which is a precursor to the binding of a loosely bound electron (lbe), calculations were first performed to describe its electronic ground state. The nuclear equilibrium geometries of the ground state and the first excited neutral as well as the ground state of the anion were calculated using the complete active space self‐consistent field (CASSCF),^[28]^ followed by the multi‐reference configuration interaction singles and doubles (MR‐CCSD) computations[^29^, ^30^] with the addition of Davidson correction for quadruples, MRCISD(Q), or with the Pople correction, MRCISD[Q]; then, the multi‐reference perturbation calculations CASPT2, the electron valence state second‐order perturbation method CAS‐NEVPT2,[^27^, ^31^, ^32^] and the multi‐reference average quadratic coupled‐cluster CAS‐AQCC method were performed.[^26^, ^33^, ^34^]

Linear equilibrium geometries of the neutral's and the anion's ground electronic states were obtained by the optimization procedures on their Born‐Oppenheimer surfaces. The reason for not using the previously reported geometries, which are all obtained by the coupled cluster techniques, is the presence of minuscule binding energies for the DBXS, which need to be calculated at geometries obtained within the same (or similar) level of theory. Dipole bound states were calculated at the equilibrium geometry of the neutral, distinctively from the previous calculations of DBXS of the C_5_N^−^ anion in EOM‐CC calculations, where the geometry of the anion was used. This is due to the fact that a loosely bound electron (lbe) attaches to the parent neutral molecule, which physically means that the core and valence molecular orbital arrangements in a DBXS is expecting to be almost equal to those for the neutral arrangement of orbitals. Moreover, previous studies (e. g., [35]) have shown that the equilibrium arrangements of the nuclei of a neutral and a corresponding DBS are almost the same, so that changes in geometry and zero‐point vibrational energies of the nuclei can be neglected.

The MOLPRO is using internally contracted procedure for MRCI, icMRCI, while in the ORCA package we used the uncontracted approach. For all CAS‐MRCI, the first 6 core orbitals were set as frozen. In SCF calculations, convergence threshold for the density matrix was set to accu=11 (10^−11^), in order to achieve convergence with very diffuse basis sets. The specific procedure we used in ORCA is described in detail in our previous work,^[17]^ so we shall not be repeating it here.

The active space for multi‐reference computations was carefully selected from the inspection of the HF orbitals and from the composition of CASSCF natural orbitals produced by various active spaces. The best choice, taking into account the best accuracy‐to‐computational‐resources ratio, for both neutral and anion calculations, consists of 13*σ*, 14*σ*, 2*π*, 3*π*, 4*π* and 5*π* orbitals, the CAS(9,10) for the neutral or CAS(10,10) level for anion. Calculations were performed in the framework of C_2v_ symmetry, specifying explicitly irreducible representations of the considered states.

To calculate the dipole‐bound states, we used the aug‐cc‐pVXZ series of valence basis sets,^[36]^ where X is the cardinal number X=2,3,4 for D, T, and Q basis sets, respectively, augmented by an additional set of even‐tempered sequences of diffuse s and p functions centered on the positive end of the parent radical, the terminal C atom. The exponents of these additional functions were determined in the same way as in our previous study.^[17]^ The diffuse functions were added to the core aug‐cc‐pVXZ basis sets, using the formula $\alpha_{n}=\alpha_{1}q^{n-1}$ ,^[35]^ n=1,2,... , where *α*_1_ is the value of the lowest exponent in the expansion and the geometrical progression ratio was set to q=5 . To select the value for *q* and the number of additional sp functions, the lowest unoccupied molecular orbital (LUMO) of the neutral Hatree‐Fock (HF) wavefunctions was analyzed in detail, in particular their energy values obtained by using different basis sets and their composition in terms of additional basis functions. If, to a first approximation, the value of the electron affinity (EA) according to the Koopmans’ theorem is equal to the negative value of the LUMO, then a negative LUMO means a positive EA, from which we can conclude that a negative very diffuse LUMO can be an option for the extra electron to be bound by the parent neutral molecule. We analyzed all the basis sets determined with the scaling factor *q* equal to 3, 3.5, 4, 4.5 and 5, with the addition of +1s1p up to the 7*s*7*p* functions; The number of terms in a sequence of diffuse exponents was selected by inspection of the energy (given in the ESI) and of the Hartree‐Fock LUMO composition, i. e. the LUMO should have as smaller energy and the *s* and *p_z_* basis functions with the lowest exponents should not dominate the LUMO of the neutral. The aug‐cc‐pVTZ+6s6p with scaling factor q=5 was chosen as the optimal basis set for the methods used in this work. On the other hand, aug‐cc‐pVXZ sets without additional diffuse basis functions were used to represent valence states of the anion and the neutral. The results of the above decisions and calculations are analysed in the following section.

One should also note here that this anion forms many valence‐bound excited states (HCN or HNC, for example, do not form these states, so the method is much simpler in this case), and if we want to calculate the lowest triplet state of the irreducible A_1_ representation for the lowest triplet sigma state, we get the lowest valence anion state of this symmetry, which is not sensitive to the additional basis diffusion functions, and moreover they have low (regular) dipole moments.

## Results and Discussion

One of the outcomes from the present work is to confirm the experimental finding which indicates the ground state of the neutral radical to be the $X^{2}\Sigma^{+}$ state, with the dominant electronic configuration of $...2\pi^{4}3\pi^{4}13\sigma^{1}14\sigma^{0}$ , where the LUMO is the 14*σ* orbital, and the highest occupied orbital (HOMO) in the CASSCF calculations is the 13*σ* orbital. The anion has the doubly occupied dominant electronic configuration of $...13\sigma^{2}2\pi^{4}3\pi^{4}14\sigma^{0}$ and the ground state $X^{1}\Sigma^{+}$ ; its electronic configuration calculated with the CASSCF(10,9)/aug‐cc‐pVQZ is 13*σ* ^1.98^ 2*π* ^3.91^ 3*π* ^3.85^ 4*π* ^0.18^ 5*π* ^0.08^, where the superscripts are the occupation numbers of the natural orbitals, which can take values from 0 to 2 for a *σ* orbital and from 0 to 4 for a *π* orbital. For the neutral radical, the CASSCF(9,9)/aug‐cc‐pVQZ electronic configuration is: 2*π* ^3.87^ 3*π* ^3.83^ 13*σ* ^1.00^ 4*π* ^0.20^ 5*π* ^0.12^. In the calculations of valence states, the LUMO 14*σ* had an occupation number below 0.02 and was therefore not included.

The electronic energies of both the neutral ground state and the bound anion ground state are shown in Table 1. We can compare the relative energies obtained in the present work by using several levels of theory with the experimentally obtained value of the EA, since we do not have a similar experimental estimate for a possible dipole bound state. Energies do not include zero‐point energy contributions.

**Table 1:** Adiabatic electron affinity (AEA) of the C_5_N/C_5_N^−^ system and permanent dipole moments *μ* (in Debye).

	Anion $X^{1}\Sigma^{+}$		Neutral $X^{2}\Sigma^{+}$		
Level of theory	*E* [a.u.]	*μ* [D]	*E* [a.u.]	*μ* [D]	AEA [eV]
CASSCF/aTZ	−243.877383	5.739	−243.778110	−3.453	2.701
CASSCF/aQZ^[a]^	−243.890466	5.735	−243.791492	−3.454	2.693
CAS‐MRCISD/aTZ	−244.566727	5.688	−244.425655	−3.427	3.839
CAS‐MRCISD/aQZ^[a]^	−244.615114	5.680	−244.473507	−3.435	3.853
CAS‐MRCISD(Q)/aTZ	−244.695086		−244.540228		4.214
CAS‐MRCISD(Q)/aQZ^[a]^	−244.748363		−244.592664		4.237
CAS‐MRCISD[Q]/aQZ^[a]^	−244.760289		−244.600941		4.336
CASPT2/aQZ	−244.756278	5.166	−244.592449	−3.375	4.458
CAS‐AQCC/aTZ	−244.673965	5.738	−244.514733	−3.325	4.333
CAS‐AQCC/aQZ	−244.751800	5.526	−244.589804	−3.425	4.408
CAS‐NEVPT2 (10, 11)/aTZ	−244.753575		−244.599481		4.193
Expt.					4.45±0.03^[b]^
Prev. theor.		5.233^[c]^		3.45^[d]^ −3.412^[e]^	4.79^[f]^ 4.50^[g]^

aTZ is an abbrev. for aug‐cc‐pVTZ and aQZ for aug‐cc‐pVQZ basis sets. [a] Geometry in the aTZ basis set of the same method. [b] Ref. [22]. [c] Ref. [12]. [d] Ref. [19]. [e] Ref. [37]. [f] Ref. [20]. [g] Refs. [13, 39].

The ground state of the neutral C_5_N radical has the same symmetry as that which we had found for the smaller radical of the same cyanopolyyne series, C_3_N, ^2^Σ^+^, Table 1, while it has a fairly large permanent electric dipole moment of about −3.4 Debye, in good agreement with previous theoretical value of −3.412 Debye obtained with the coupled‐cluster approach.^[37]^ Important for this work is that this dipole moment is larger than the critical value of about 2 Debye,^[38]^ and is therefore able to attach the free electron via its dipole field, thereby forming a dipole‐supported anionic electronic state. Consequently, we expect that calculations such as those previously performed for the C_3_N^−^ anion^[17]^ would also allow us to locate and confirm the presence of dipole‐bound states for the larger C_5_N^−^ anion.

The values for the electron affinity of the neutral C_5_N, which are determined in this work to be close to the experimental value^[22]^ of 4.45±0.03 eV, were obtained by CAS‐AQCC/aug‐cc‐pVQZ and CASPT2/aug‐cc‐pVQZ procedures. The MRCISD values turn out to be too low and need to be corrected with the quadratic Q contribution, i. e., either the Davidson MRCISD(Q) or the Pople corrections MRCISD[Q], to approach the value of 4.4 eV.

The two sets of geometries determined by CAS‐MRCISD(Q) and CAS‐AQCC are presented in Table 2, where AQCC values differ slightly from the previously obtained CCSD(T)/aug‐cc‐pVQZ bond lengths, recommended by Botschwina (shown in the footnote to Table 2),^[12]^ with the root‐mean‐square‐deviation value (RMSD) of 0.006 Angstroms for the Σ state of the neutral. The MRCI(Q) geometry for the same state has RMSD value of 0.009 Angstroms in respect to the same previously calculated theoretical geometry,^[12]^ which is less than 10^−12^ m.

**Table 2:** Equilibrium nuclear geometry (in Angstroms) of the electronic states and equilibrium rotational constants (in MHz).

	Anion $X^{1}\Sigma^{+}$		Neutral $X^{2}\Sigma^{+}$		Neutral $A^{2}\Pi$	
	MRCI/aTZ^[a]^	AQCC/aQZ^[a]^	MRCI/aTZ	AQCC/aQZ	MRCI/aTZ	AQCC/aQZ
$r_{C1C2}$	1.2556	1.2599	**1.2110**	1.2130	1.2921	1.2971
$r_{C2C3}$	1.3453	1.3471	**1.3722**	1.3732	1.3470	1.3432
$r_{C3C4}$	1.2266	1.2317	**1.1943**	1.2060	1.2022	1.2166
$r_{C4C5}$	1.3629	1.3648	**1.3768**	1.3788	1.3712	1.3722
$r_{C5N}$	1.1498	1.1629	**1.1575**	1.1616	1.1589	1.1634
*B_e_* ^[b]^	1394	1384	1402	1394	1388	1379
*B_e_* prev. theor.	1389.4^[c]^		1397±3^[d]^ 1421^[e]^		1385^[d]^	
*B_o_* expt.	1388.9^[f]^		1403^[g]^			

[a] MRCI is an abbrev. for CAS(9/10,9)‐MRCISD(Q), AQCC for CAS‐AQCC, aTZ for aug‐cc‐pVTZ, aQZ for aug‐cc‐pVQZ. [b] Calculated with average atomic masses. [c] Ref. [12], with recommended geom. of 1.2582, 1.3453, 1.2313, 1.3570, and 1.1698. [d] Ref. [39]. [e] Ref. [44]. [f] Ref. [4]. [g] Ref. [23]. Prev. theor. recommended geometry for the neutral X^2^Σ^+^: 1.2136, 1.3654, 1.2124, 1.3710, 1.1619, and for A^2^Π state 1.2961, 1.3283, 1.2298, 1.3624, 1.1640, Ref. [37]

The equilibrium rotational constants B_*e*_ are summarized in Table 2 for each geometry, together with the previously determined theoretical B_*e*_ and experimental B_0_ values. The equilibrium B_*e*_ value can provide a good estimate for the measured rotational constant B_0_. The most recent microwave detection of the anion in TMC‐1^[4]^ reported a very precise value of 1388.86681(19) MHz for the ground anion, very close to the previously recommended value of 1389.4 MHz.^[12]^ For the neutral ground state, the experimental value of 1403 MHz^[23]^ agrees with the MRCI value of 1402 MHz found in the present study, supporting this geometry. The previously determined theoretical value of 1397±3 MHz^[39]^ is slightly smaller than the latter value. As far as we can find, there are no experimental data for the rotational constant of the excited neutral ^2^Π state; the previously reported value of 1385 MHz^[39]^ is close to the MRCI result of 1388 MHz. Based on these results, the MRCI(Q) equilibrium nuclear geometry of the neutral was used for the calculations of the dipole‐bound excited states of the anion.

The equilibrium permanent dipole moments, *μ*, of about 5.5 D for the ground anion and of −3.4 Debye for the ground neutral, were evaluated in the center‐of‐mass coordinate system and defined to have positive sign when directed from negative to positive values along the *z* axis; The internuclear *z* axis is in the direction of the terminal C_1_ and towards N. Therefore, a minus sign for the neutral means that the dipole is oriented in the opposite direction, i. e. from N to C_1_, so the positive part of the dipole that attracts the electron is on the terminal carbon. As we can see, the radical undergoes significant change of its dipole moment from −3.4 to +5.5 D (overall about 9 Debye) when attaches an excess electron and relaxes its structure to that of the anion's ground state.

All calculations in this work have shown (see Table 3) that the ^2^Π state of the neutral is above the ^2^Σ^+^ state. This state has the $...13\sigma^{2}$ $2\pi^{4}$ $3\pi^{3}$ $14\sigma^{0}$ dominant electronic configuration (instead of the partially filled 13*σ* orbital as in the ground ^2^Σ^+^ state, the 3π here has 3 electrons). An important fact for this work on DBSs is that the ^2^Π state has very small permanent dipole moment. For example, the CAS‐AQCC/aug‐cc‐pVQZ wavefunction at the PES minimum of this state gives −0.138 Debye, and CAS‐MRCISD(Q)/aug‐cc‐pVQZ yielded the even smaller value of −0.077 Debye. This means that the $A^{2}\Pi$ state of the neutral cannot produce a large enough dipole field and consequently cannot bind an excess electron to produce a bound DBS.

**Table 3:** Energy of the *A* ^2^Π state of the neutral, its permanent dipole moment *μ* (in Debye) and adiabatic excitation energy from the *X* ^2^Σ^+^.

	Neutral $A^{2}\Pi$		
Level of theory	*E* [a.u.]	*μ* [D]	ΔE [eV]
CAS‐MRCISD/aTZ	−244.411783	−0.053	0.377
CAS‐MRCISD/aQZ^[a]^	−244.458384	−0.077	0.412
CAS‐MRCISD(Q)/aTZ	−244.532407		0.213
CAS‐MRCISD(Q)/aQZ^[a]^	−244.582830		0.268
CAS‐MRCISD[Q]/aQZ^[a]^	−244.592106		0.240
CAS‐AQCC/aQZ	−244.582116	−0.138	0.209
Prev. theor.		0.11^[b]^	0.06±0.02^[c]^
Expt. TOF‐PE^[d]^			0.069±0.015

aTZ is an abbrev. for aug‐cc‐pVTZ and aQZ for aug‐cc‐pVQZ basis sets. [a] Geometry in TZ basis set. [b] Ref. [20] [c] Ref. [39], in cm^−1^ 500±200. [d] Ref. [22], in cm^−1^ 560±120.

In the previous theoretical work on the possible DBSs of the C_5_N^−^ anion, Skomorowski et a.l^[20]^ found that the ground state of this radical is the ^2^Π state, so no bound DBS were predicted by their calculations. “We did not find any DBS in other cyanopolyynes (CN^−^, C_5_N^−^ and C_7_N^−^), which is consistent with the low values of dipole moments of the respective neutral precursors in their electronic ground states.”^[20]^ However, the same assignment of the ground state ^2^Σ^+^ as in this work has been confirmed both experimentally^[22]^ and theoretically.^[12]^

In our calculations on the possible existence of DBS in the C_5_N^−^ anion, we could not take into account the excitation of an electron from the ground state of this anion, which has the 3*π* orbital as HOMO, as would be required for the prediction of the excited valence states of this species. This is because the initial *π* ^3^ electronic configuration would yield the Π electronic state of the excited anion when an electron is promoted to the 14*σ* LUMO. Instead, the promotion from the 13*σ* orbital of the anion to the 14*σ* is investigated, looking for the DBS with the electronic configuration $...2\pi^{4}3\pi^{4}13\sigma^{1}14\sigma^{1}$ . The neutral radical with its dipole field has the partially occupied 13*σ* orbital consisting mainly of the 2p_*z*_ orbital of the terminal carbon atom. The doubly occupied 13*σ* orbital, on the other hand, has a lower energy than all *π* orbitals of the anion in the ground state, and the transition from this orbital to the excited orbitals does not occur at lower excited states of the anion. Therefore, the arrangement of the molecular orbitals in the neutral state corresponds more to the DBS anion than to the ground state of the anion, which is also the reason for calculating the DBS in the neutral geometry.

**Figure 1:**
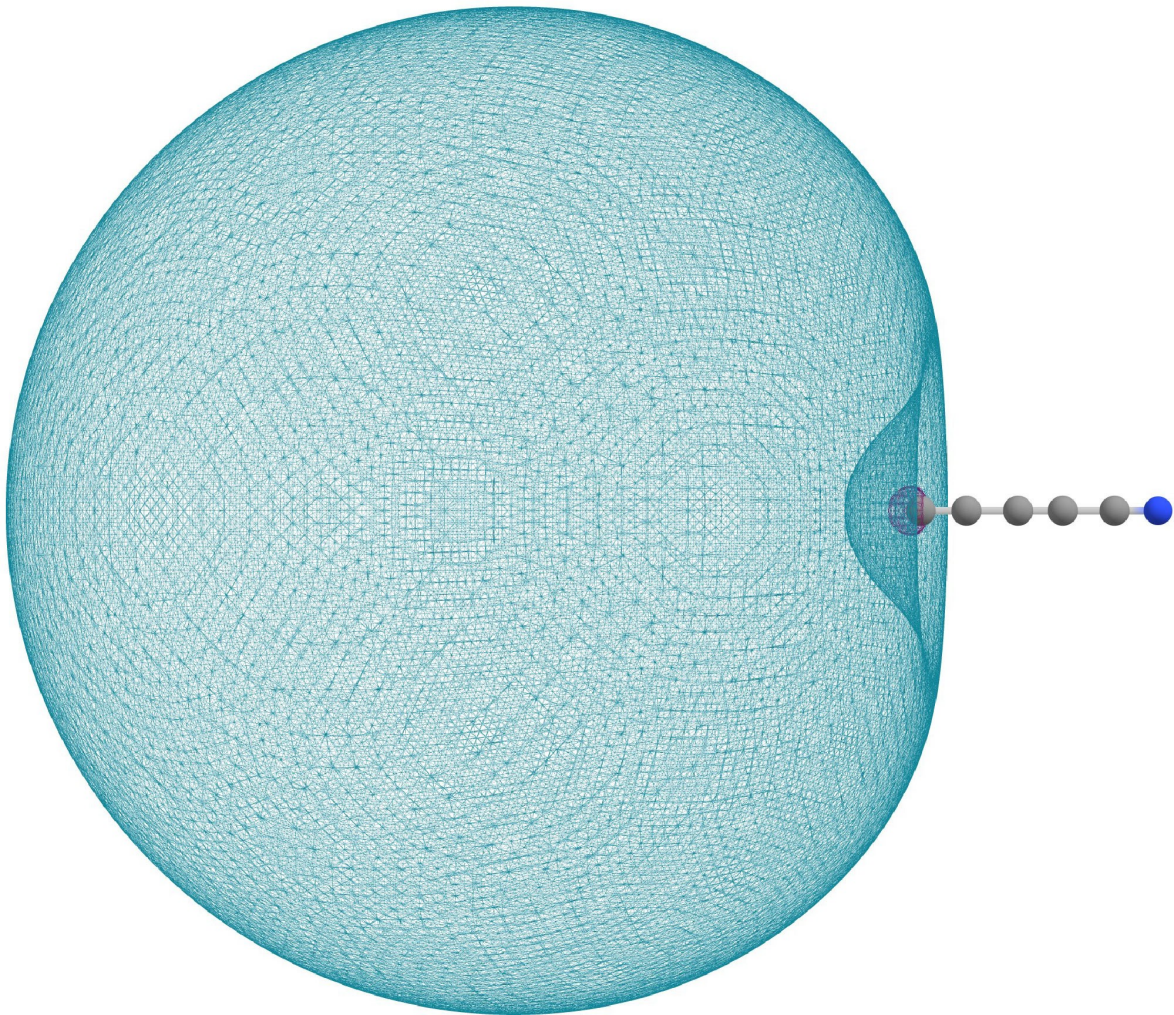
The HOMO orbital of the ^3^Σ^+^ dipole bound state (The electron density isovalue is of 0.0011 a.u.).

Table 4 summarises the electron binding energies (EBE) of the DBXSs ^1^Σ^+^ and ^3^Σ^+^ arising from the same electronic dominant configuration $...2\pi^{4}3\pi^{4}13\sigma^{1}14\sigma^{1}$ in the neutral geometry using multireference methods for excited states. The vertical energy difference $E_{neutral}-E_{anion}$ corresponds to the electron binding energy (EBE). Using the wave function obtained from the MRCISD(Q)/aug‐cc‐pVTZ+6s6p calculations, a very diffuse 14*σ* orbital located on the terminal carbon atom is obtained (see Figure 1).

**Table 4:** Two excited dipole‐bound states ^3^Σ^+^ and ^1^Σ^+^ of C_5_N^−^ anion calculated with +6s6p diffuse functions.

Level of theory	*E_n_* $\left(X^{2}\Sigma^{+}\right)$ [a.u]	*E_a_* 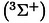 [a.u]	EBE [cm^−1^]	*E_a_* 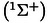 [a.u]	EBE [cm^−1^]
CAS(10,11)‐MRCISD(Q)/aTZ+^[a]^	−244.578237	−244.578279	9.22	−244.578251	3.07
CAS(6,11)‐MRCISD(Q)/aTZ+^[b]^	−244.578237	−244.578280	9.39	−244.578250	2.85
icMRCISD/CBS+^[c]^	−244.418820	−244.418863	9.48		
icMRCISD(Q)/CBS+^[c]^	−244.576140	−244.576181	9.11		
icMRCISD[Q]/CBS+^[c]^	−244.591267	−244.591308	8.98		
CASPT2/aTZ+^[a]^)	−244.530032	−244.530077	9.77		
CAS(10,11)‐NEVPT2/CBS+^[a]^	−244.786298	−244.786335	8.10	−244.786309	2.48

CBS is an abbrev. for complete basis set, aTZ for aug‐cc‐pVTZ, aQZ for aug‐cc‐pVQZ. [a] Active space 13σ2π3π4π5π14σ15σ . [b] Active space 13σ3π4π5π14σ15σ16σ17σ . [c] One selective configuration $13\sigma^{1}14\sigma^{1}2\pi^{4}3\pi^{4}4\pi^{0}5\pi^{0}$ .

The MRCI(Q) method yields consistent values of about 3 and 9 cm^−1^ for the ^1^Σ^+^‐ and ^3^Σ^+^‐excited dipole‐bound states, respectively; in addition, the NEVPT2 and CASPT2 perturbational methods yield energies that are close to the MRCI(Q) results. The CAS‐AQCC method implemented in MOLPRO 2012, which performed best when carrying out the AEA calculations for the electronic ground states, is not recommended and supported for use in excited state calculations in MOLPRO 2012.1.^[26]^

In Table 5, 6, EBE values for the ^3^Σ^+^ state are obtained for several levels of theory using the complete basis set (CBS) limit energy values. The CBS energies are estimated from the correlation‐consistent aDZ, aTZ and aQZ basis set sequence augmented with +6 s6p diffusion functions (q=5) using the exponential extrapolation scheme $E\left(X\right)=E_{lim}+Bexp\left(-\alpha X\right)$ , where *X* is the cardinal number 2, 3, 4 for aDZ, aTZ and aQZ, respectively.[^40^, ^41^] The values obtained range from 8.1 to 9.5 cm^−1^ and are shown in Table 4. To check the reliability of this extrapolation scheme in this case, we fitted the icMRCISD energies (the first row in Table 5) into the power function form $E\left(X\right)=E_{lim}+BX^{-\alpha }$ ,[^40^, ^41^, ^42^] and obtained the formula $E\left(X\right)=E_{lim}+1.4644X^{-2.5267}$ for both the neutral and triplet DBXS and obtained an EBE of 9.50 cm^−1^, which is very close to the 9.48 from the exponential fit. Despite a possible large extrapolation error due to the omitted a5Z energy values, the results are quite consistent.

**Table 5:** Electron binding energy (EBE) of the DBXS ^3^Σ^+^, determined by the use of the CBS limit values.

Method		aDZ+6s6p	aTZ+6s6p	aQZ+6s6p	CBS limit	EBE [cm^−1^]
icMRCISD^[a]^	N	−244.189614	−244.352512	−244.399637	−244.418820	9.48
	A	−244.189653	−244.352553	−244.399679	−244.418863	
icMRCISD(Q)^[a]^	N	−244.320884	−244.504097	−244.555807	−244.576140	9.11
	A	−244.320922	−244.504137	−244.555848	−244.576181	
icMRCISD[Q]^[a]^	N	−244.334674	−244.519571	−244.571234	−244.591267	8.98
	A	−244.334712	−244.519611	−244.571274	−244.591308	
CAS(10,11)‐NEVPT2^[b]^	N	−244.307626	−244.599481	−244.713387	−244.786298	8.10
	A	−244.307692	−244.599542	−244.713438	−244.786335	

N is an abbrev. for the neutral $X^{2}\Sigma^{+}$ and A for the anion ^3^Σ^+^, aDZ for aug‐cc‐pVDZ, aTZ for aug‐cc‐pVTZ, aQZ for aug‐cc‐pVQZ. Energy is in a.u. [a] Active space $13\sigma^{1}14\sigma^{1}2\pi^{4}3\pi^{4}4\pi^{0}5\pi^{0}$ . [b] Active space 13σ3π4π5π14σ15σ16σ17σ .

**Table 6:** Electron binding energy (EBE) of the DBXS ^3^Σ^+^, determined by the aug‐cc‐pVTZ basis set with the addition of diffuse functions.

Method		+4s4p	+5s5p	+6s6p	+7s7p	+6s6pdf	+6s6p6d
icMRCISD	N	−244.352512	−244.352512	−244.352512	−244.352512	−244.352644	−244.352559
	A	−244.352534	−244.352553	−244.352553	−244.352553	−244.352685	−244.352602
	EBE	4.87	9.00	9.02	9.02	9.04	9.42
icMRCISD(Q)	N	−244.504097	−244.504097	−244.504097	−244.504097	−244.504249	−244.504150
	A	−244.504117	−244.504137	−244.504137	−244.504137	−244.504289	−244.504192
	EBE	4.30	8.71	8.72	8.72	8.80	9.08
icMRCISD[Q]	N	−244.519571	−244.519571	−244.519571	−244.519571	−244.519724	−244.519625
	A	−244.519590	−244.519611	−244.519611	−244.519611	−244.519764	−244.519666
	EBE	4.16	8.63	8.65	8.65	8.72	8.97

N is an abbrev. for the neutral $X^{2}\Sigma^{+}$ , A for the anion ^3^Σ^+^. EBE is in cm^−1^, electronic energies in a.u.

In Table 6 the EBE values for the excited state ^3^Σ^+^ are presented, this time by changing additional diffuse functions in the aug‐cc‐pVTZ basis set from +4s4p to +7s7p. Only +4s4p is not large enough for the description of this DBXS. With the addition of even more diffuse functions with lower exponents, the energy of the DBS converges to one value and the EBE is 9.02, 8.72 and 8.65 for the methods presented. Furthermore, diffuse functions with larger angular momentum are added in the last two columns of Table 6. The 6d functions give slightly higher values for EBE, but all energies are around 9 cm^−1^.

In Table 7 we show the LUMO Hartree‐Fock energies and EBE_*KT*_, obtained as −E_*LUMO*_ according to Koopmans’ Theorem, for scaling factor q=5. Convergence is achieved even for +5s5p functions. The EBE for this state is about 4.8 cm^−1^, and correlation effects increase this value to about 9 cm^−1^.

**Table 7:** LUMO HF energies and EBE_*KT*_ for the neutral *X* ^2^Σ^+^ state

q=5.0		

Basis set	LUMO Energy [a.u.]	EBE_*KT*_ [cm^−1^]
aug‐cc‐pVTZ	0.039493	0
+3s3p	0.000132	0
+4s4p	−0.000012	2.63
+5s5p	−0.000022	4.83
+6s6p	−0.000022	4.83
+7s7p	−0.000022	4.83

For the singlet ^1^Σ^+^ state, there were problems with CI convergence when using such a diffuse set of functions where root flipping for A_1_ symmetry was common. However, the results obtained with both NEVPT2 and MRCI(Q) are around 3 cm^−1^.

Although the parent molecule C_5_N has a considerably large dipole moment, the binding energies are extremely small. For the closely related anion HC_5_N^−^, for example, we obtained an EBE of about 130 cm^−1^ for the ground state DBS, which is the doublet state ^2^Σ^+^.^[43]^ Our previous theoretical and experimental results on C_3_N^−^ suggest that the MRCI results agree well with the experimental value of 2 cm^−1^.[^16^, ^17^] These values are even lower than for HCN^−^, which has a calculated EBE value of about 10 cm^−1^.^[21]^ These small values can be attributed to the open‐shell character of the neutral parent radical.^[20]^

## Present conclusions

In the present work, we have used the multireference electron structure methods at a high level, which we have reported in detail in the previous section The computational tools. We have therefore shown that the neutral radical C_5_N, already discovered in interstellar space as reported in the introductory section, is capable of weakly binding an extra electron through its electric dipole field. The loosely bound excess electron can in turn form two possible excited bound states with the same electronic configuration very close to the electron detachment threshold: the singlet ^1^Σ^+^ and a triplet ^3^Σ^+^, both physically dipole bound states with large electric dipole moments. The EBEs obtained are in the range of 8.1 to 9.5 cm^−1^ for the ^3^Σ^+^ and 2.5 to 3.1 cm^−1^ for the ^1^Σ^+^ DBXS. These are low binding energies, so this anion can be classified as a very weakly bound excited electronic state.^[38]^

The present results represent the first theoretically estimated values for such loosely bound anionic states of the title molecule, while no experimental studies of the DBS states have yet been reported for this anion. The binding energy value of 3 cm^−1^ for the singlet state to which the dipole transition from the singlet ground state is allowed turns out to be very similar to the value obtained in earlier experiments on the smaller cyanopolyyne of the series, C_3_N, where we found a value of about 2 cm^−1^.^[16]^ It is expected that the presence of a DBXS anion in C_5_N^−^ also affects its electronic spectrum. It is interesting to note that earlier scattering calculations on the C_3_N^−^ and the C_2_H^−^ species^[45]^ indicated the presence of a weakly bound state in the former anion by about 9.7 cm^−1^, confirming the difficulty of obtaining consistent values for such special, bound anionic states.

In our present work, we estimate a permanent dipole moment of about 5.5 Debye for the ground state C_5_N^−^ anion, of −3.4 for the neutral $X^{2}\Sigma^{+}$ in the ground state, and the small value of −0.1 Debye for the $A^{2}\Pi$ anion. Since we also found that the neutral $A^{2}\Pi$ is about 0.28 eV above the ground state, the dipole moment of the radical in the ISM should be closer to the ground state value and not to the mean value of the two neutral states, which explains the calculated abundance ratio in Ref. [4].

All the multi‐reference methods employed in this work predict that the adiabatic excitation energy of the $A^{2}\Pi$ state from the ground state $X^{2}\Sigma^{+}$ of the radical is larger than 0.2 eV. These values are significantly different from the previously determined energy of about 0.06 eV (500 cm^−1^).[^22^, ^39^] Although we cannot fully explain this discrepancy, this relatively small excitation energy means that, when calculating rovibrational states in the neutral, nonadiabatic effects should be taken into account since they could be made active via vibronic coupling between such nearby electronic states through the bending modes that have *π* symmetry.

In the quantum theoretical treatment of anion formation by REA by Khamesian et al,,^[13]^ they concluded that their REA cross sections were too small to explain the formation of C_5_N^−^ in the ISM by that direct process, although the possibility of forming intermediate weakly bound DBSs was not considered. More recently, model calculations have been carried out for the same REA process but involving instead only the capture into a DB state of the C_3_N molecule.^[46]^ Their model calculations did not include correctly the exchange interaction, using instead a well‐known DFT model exchange. Their findings obtained capture cross sections into the DB states which were even smaller than those of reference^[13]^ by several orders of magnitude, thereby arguing that even capture into a DB bound state would be of marginal importance.

Because of the lack of exact exchange and correlation effects on the above calculations it is however hard to assess their accuracy. That the existence of DBS anions could provide possible gateways for the attachment of threshold electrons is therefore still an open question and it remains to be proven whether they could or not increase the cross sections into the VB states enough to explain the fairly large abundance of this anionic cyanopolyyne in the ISM. The alternative chemical routes thus provide viable paths to the formation of this anion and were specifically considered in our earlier work,^[15]^ where we have found them to provide reactive rate coefficients much larger than the REA coefficients computed thus far and therefore were found to be one of the more likely mechanisms to the anion formation at the low temperatures of that ISM environment.

Previous calculations, all based on the coupled cluster methodology, showed that the ground states of the neutral C_7_N and C_9_N are both ^2^Π states that have very small permanent dipole moment,[^12^, ^18^, ^19^, ^20^] so these species are not considered likely to form DBS anions. However, these larger cyanopolyynes could also be of interest for later studies.

## Conflict of interest

There are no conflict to declare.

1

## Supporting information

As a service to our authors and readers, this journal provides supporting information supplied by the authors. Such materials are peer reviewed and may be re‐organized for online delivery, but are not copy‐edited or typeset. Technical support issues arising from supporting information (other than missing files) should be addressed to the authors.

Supporting Information

## Data Availability

The data that support the findings of this study are available in the supplementary material of this article.
