# Supplementary material for: Investigating Possible Dipole‐Bound States of Cyanopolyynes: the Case for the C5N− Anion Detected in Interstellar Space
Source: Chemphyschem. 2023 Oct 24;24(22):e202300248. doi: 10.1002/cphc.202300248 (PMC10962575; doi:10.1002/cphc.202300248)
Supplement: Supplementary file 1 — Supporting Information [file CPHC-24-0-s001.pdf]

ChemPhysChem

Supporting Information

Investigating Possible Dipole-Bound States of Cyanopolyynes: the Case for the C_5N^- Anion Detected in Interstellar Space

Stanka Jerosimić,* Milan Milovanović, Marko Mitić, Roland Wester, and Francesco A. Gianturco*

Electronic supplementary material (ESI)

Table S1. Hatree-Fock results for different basis sets and q=3.0

	$q = 3.0$		
Basis set	Dipole moment [Debye]	SCF Energy [a.u.]	LUMO Energy [a.u.]
aug-cc-pVTZ	3.56212	−243.653430	0.039493
+3s3p	3.56182	−243.653445	0.001121
+4s4p	3.56182	−243.653446	0.000244
+5s5p	3.56182	−243.653446	0.000031
+6s6p	3.56182	−243.653446	−0.000015
+7s7p	3.56182	−243.653446	−0.000022

Table S2. Hatree-Fock results for different basis sets and q=3.5

	$q = 3.5$		
Basis set	Dipole moment [Debye]	SCF Energy [a.u.]	LUMO Energy [a.u.]
aug-cc-pVTZ	3.56212	−243.653430	0.039493
+3s3p	3.56190	−243.653441	0.000631
+4s4p	3.56190	−243.653441	0.000092
+5s5p	3.56190	−243.653441	−0.000008
+6s6p	3.56190	−243.653441	−0.000021
+7s7p	3.56190	−243.653441	−0.000022

Table S3. Hatree-Fock results for different basis sets and q=4.0

	$q = 4.0$		
Basis set	Dipole moment [Debye]	SCF Energy [a.u.]	LUMO Energy [a.u.]
aug-cc-pVTZ	3.56212	−243.653430	0.039493
+3s3p	3.56195	−243.653438	0.000367
+4s4p	3.56195	−243.653438	0.000029
+5s5p	3.56195	−243.653438	−0.000019
+6s6p	3.56195	−243.653438	−0.000022
+7s7p	3.56195	−243.653438	−0.000022

Table S4. Hatree-Fock results for different basis sets and q=4.5

	$q = 4.5$		
Basis set	Dipole moment [Debye]	SCF Energy [a.u.]	LUMO Energy [a.u.]
aug-cc-pVTZ	3.56212	−243.653430	0.039493
+3s3p	3.56199	−243.653436	0.000219
+4s4p	3.56199	−243.653436	0.000001
+5s5p	3.56199	−243.653436	−0.000021
+6s6p	3.56199	−243.653436	−0.000022
+7s7p	3.56206	−243.653436	−0.000022

Table S5. Hatree-Fock results for different basis sets and q=5.0

	$q = 5.0$		
Basis set	Dipole moment [Debye]	SCF Energy [a.u.]	LUMO Energy [a.u.]
aug-cc-pVTZ	3.56212	−243.653430	0.039493
+3s3p	3.56201	−243.653435	0.000132
+4s4p	3.56201	−243.653435	−0.000012
+5s5p	3.56201	−243.653435	−0.000022
+6s6p	3.56201	−243.653435	−0.000022
+7s7p	3.56153	−243.653435	−0.000022
